# Mutation-Specific Mechanisms of Hyperactivation of Noonan Syndrome SOS Molecules Detected with Single-molecule Imaging in Living Cells

**DOI:** 10.1038/s41598-017-14190-6

**Published:** 2017-10-26

**Authors:** Yuki Nakamura, Nobuhisa Umeki, Mitsuhiro Abe, Yasushi Sako

**Affiliations:** Cellular Informatics Laboratory, RIKEN, 2-1 Hirosawa, Wako, 351-0198 Japan

## Abstract

Noonan syndrome (NS) is a congenital hereditary disorder associated with developmental and cardiac defects. Some patients with NS carry mutations in SOS, a guanine nucleotide exchange factor (GEF) for the small GTPase RAS. NS mutations have been identified not only in the GEF domain, but also in various domains of SOS, suggesting that multiple mechanisms disrupt SOS function. In this study, we examined three NS mutations in different domains of SOS to clarify the abnormality in its translocation to the plasma membrane, where SOS activates RAS. The association and dissociation kinetics between SOS tagged with a fluorescent protein and the living cell surface were observed in single molecules. All three mutants showed increased affinity for the plasma membrane, inducing excessive RAS signalling. However, the mechanisms by which their affinity was increased were specific to each mutant. Conformational disorder in the resting state, increased probability of a conformational change on the plasma membrane, and an increased association rate constant with the membrane receptor are the suggested mechanisms. These different properties cause the specific phenotypes of the mutants, which should be rescuable with different therapeutic strategies. Therefore, single-molecule kinetic analyses of living cells are useful for the pathological analysis of genetic diseases.

## Introduction

Noonan syndrome (NS) is a congenital genetic disorder causing cardiac and developmental defects^1,2^. The phenotypes often observed in NS are ambiguous and varied, including short stature, distinctive facial characteristics, learning problems, and a predisposition to leukemia^1,3,4^. Mutations in the SHP2, SOS, RAS, and RAF proteins have been identified in NS patients^3,5^, suggesting that NS is caused by the excessive activation of the RAS–MAPK pathway. Because this pathway plays crucial roles in various cellular phenomena^6–8^, different single mutations in one protein produce various phenotypes^9^, and the variation in phenotypes has hindered the development of medical treatments for NS. At the present time, only symptomatic treatments are available for NS patients. Clarification of the pathological mechanism of each NS mutant in hyperactivating the RAS–MAPK pathway is crucial to establishing techniques for the rational treatment of NS.

Mutations in SOS (SOS1) occur in 8–14% of all NS patients who have been genetically analyzed^10–13^. SOS is a guanine exchange factor (GEF) for the small GTPase RAS^14^. In the resting state of cells, SOS forms a complex with GRB2 in its C-terminal GRB2-binding domain and is located in the cytoplasm^14^. Upon stimulation of the receptor tyrosine kinases (RTKs) on the plasma membrane, including the epidermal growth factor (EGF) receptor (EGFR), the phosphorylation of tyrosine in the cytoplasmic domains of the RTKs is recognized by the SH2 domain of GRB2, resulting in the translocation of the GRB2–SOS complex from the cytoplasm to the plasma membrane^14^. On the plasma membrane, SOS interacts with the inactive GDP-bound form of RAS (RAS–GDP) to stimulate the exchange of RAS-bound GDP for cytoplasmic GTP, which activates RAS^14^.

SOS consists of six domains^14,15^. The N-terminal histone fold (H domain) and the third domain, the pleckstrin homology (PH) domain, bind acidic lipids. The second domain is the Dbl-homology (DH) domain. The fourth domain, the RAS-exchange motif (REM) domain, binds to RAS–GTP. The fifth domain, the Cdc25 domain, corresponds to the GEF activity and therefore binds to RAS–GDP. The last domain is the C-terminal GRB2-binding (G) domain. Therefore, SOS contains five direct and indirect membrane-associating domains (H, PH, REM, Cdc25, and G) that are involved in its translocation to the membrane. A conformational change in the SOS structure and a positive feedback loop between RAS and SOS are important in the regulation of SOS activity, in addition to its association with multiple membrane components^16,17^. The intramolecular association between the H domain and the helical linker (HL) between the PH and REM domains, stabilizes the SOS structure in the closed form^15,18,19^. In this form, the interaction between the REM domain and RAS–GTP is prevented^15,20^. When SOS takes the open form and RAS–GTP binds to the REM domain, the GEF activity of SOS in the Cdc25 domain is strongly increased^15,18^, forming an SOS–RAS positive feedback loop.

NS mutations have been reported in all six domains and the HL of SOS^14,21^. Mutations in the Cdc25 domain may directly increase the GEF activity of SOS, inducing the hyperactivation of the RAS–MAPK pathway. However, mutations in the other domains must affect RAS–MAPK activation through indirect mechanisms. Some of these indirect mutations have been classified into two classes based on the location of the mutation^21^. Some mutations in the H and PH domains are thought to increase the affinity of SOS for the lipid components of the membrane, and others are thought to destabilize the closed conformation, as suggested from the crystal structure^15^. However, these possibilities have not been examined experimentally in the context of living cells, and the molecular mechanisms by which some mutations cause NS are largely unknown.

A single-molecule kinetic analysis^22^ is a useful way to examine SOS functions in living cells^23^. Although the translocation of SOS is infrequent in single living cells, its dynamics can be visualized clearly because the single-molecule imaging of SOS molecules conjugated with a fluorophore is highly sensitive. Single-molecule imaging also allows the analysis of the association and dissociation kinetics between SOS and the membrane components^23^. We found that the simultaneous association of the H and G domains to the plasma membrane is critical for generating an intermediate dissociation state of SOS on the plasma membrane. In the intermediate state, the SOS molecule seems to adopt an open conformation, which is stabilized by the function of the REM domain^23^. SOS activity simultaneously requires intact H, G, and REM domains, suggesting that in the intermediate state the autoinhibition of SOS in the closed state is released to complete the positive feedback loop with RAS^23^.

**Figure 1:**
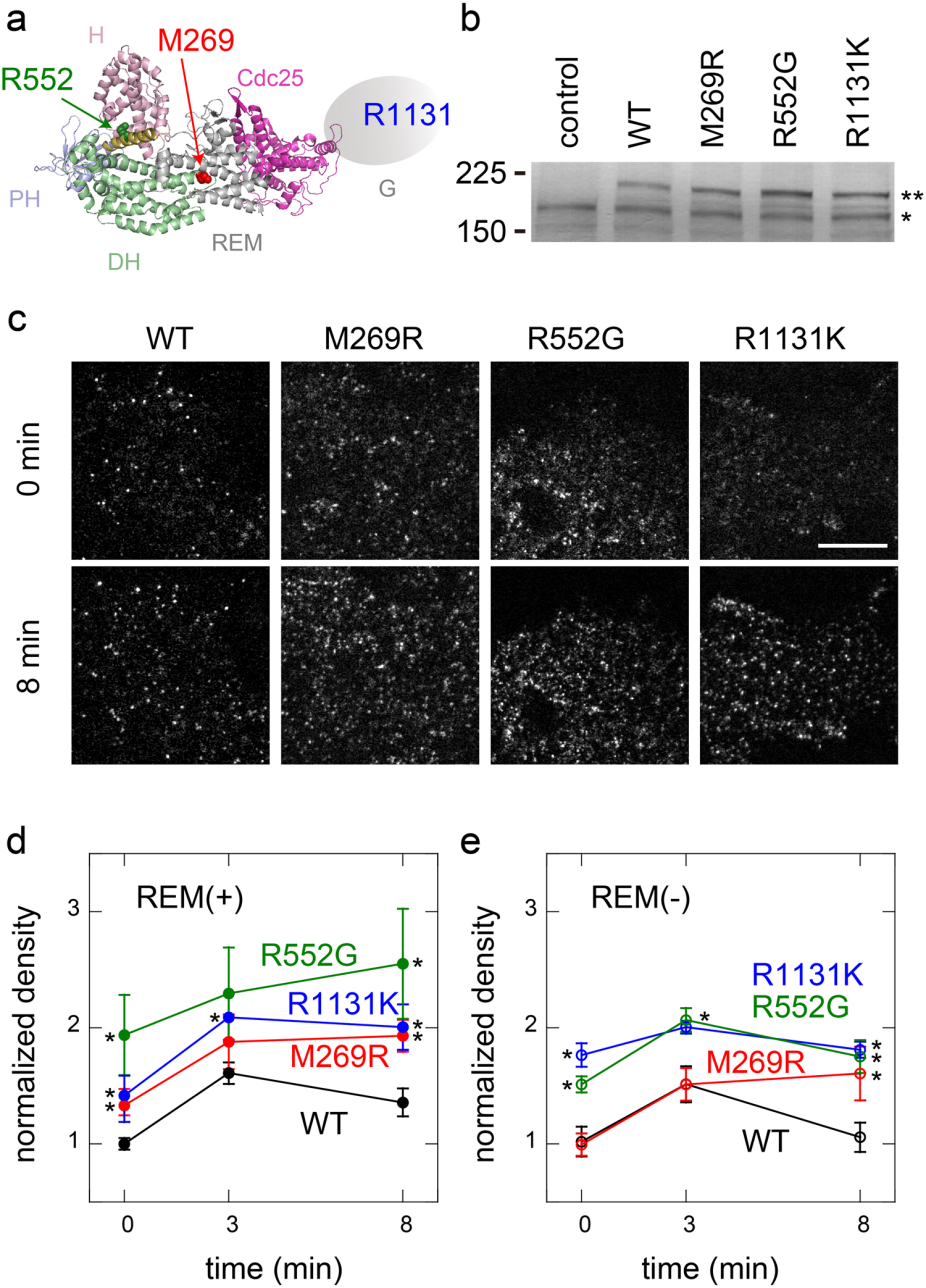
Single-molecule imaging of NS SOS dynamics. (**a**) Locations of NS mutations in SOS (M269, R552, and R1131) in the atomic structure (PDB ID: 3KSY^15^). Atomic structure of the G domain is unknown. (**b**) Expression of SOS proteins detected by immunoblotting analysis with anti-SOS1 antibody. (**Halo-tagged and *endogenous SOS). Full-length blot image is shown in Supplementary Fig. S2. (**c**) Typical micrographs of single SOS molecules on the cell surface before and after EGF stimulation for 8 min. The upper and lower micrographs show the same cells. Bar: 10 μm. (**d**,**e**) Translocation of SOS molecules to the cell surface. The molecules contained the REM(−) mutation (**e**) or did not (**d**). Densities of the fluorescent SOS molecules on the cell surfaces were normalized to the fluorescence intensity in the cytoplasm and plotted as values relative to that of WT SOS before EGF stimulation (time 0). Means of 6–10 cells are plotted with standard errors (SE). Asterisks indicate statistical significance relative to WT (**d**) or WT REM(−) (**e**) at the indicated times (p < 0.05 on Mann–Whitney test).

In this study, we analysed the single-molecule kinetics of the membrane interactions of three NS SOS mutants (M269R, R552G, and R1131K) and compared them with that of the wild type (WT). These three mutations occur in the DH, HL, and G domains, respectively^21^ (Fig. 1a). In the X-ray crystallographic structure of SOS, M269 interacts with the REM domain and is probably involved in the inhibition of the positive feedback loop with RAS^18^. R552 interacts with D140 and D169 in the H domain (in the X-ray crystallographic structure) and is thought to maintain the autoinhibitory closed structure^15^. How R1131K causes NS is unknown. Because R1131 is located in the proline-rich region of the G domain^21^, which is used for the formation of the SOS–GRB2 complex, it may change the interaction with activated RTKs via GRB2. In addition to these molecules with single-point mutations and WT, we used the corresponding molecules with an additional defect in the REM domain (the L687E/R688A/W729E triple mutant; REM(−)^18,23^) to assess the roles of the positive feedback reaction with RAS. Because the feedback reaction acts when SOS takes the open conformation^23^, comparisons with the REM(−) mutants should provide information on the SOS conformation. We found that the three NS mutations have unique effects on the basic processes of the membrane interaction.

## Results

### Cell-surface translocation of NS SOS mutants

Three different types of NS SOS mutants, M269R, R552G, and R1131K, tagged with the Halo protein at their N-termini, were expressed in HeLa cells (Fig. 1b) and stained with the tetramethylrhodamine-conjugated Halo ligand. EGF-induced ERK phosphorylation in Halo-tagged SOS knock-in HeLa cell lysate indicated that Halo-tag moiety of the fusion protein did not influence the SOS functions significantly (Supplementary Fig. S1). As we have reported previously for WT SOS^23^, single molecules of SOS were observed on the plasma membranes of the cells before and after stimulation with EGF (Fig. 1c). Little number of fluorescent particles were observed in the cells that did not express SOS molecules but were stained with the Halo ligand^23^. After the cells were stimulated with a saturating concentration of EGF (100 ng/ml), the densities of the NS SOS molecules on the cell surfaces (normalized to the concentrations in the cytoplasm) were increased (Fig. 1d). Compared with the translocation dynamics of WT, all three NS mutants displayed hypertranslocation. The normalized densities were higher for the NS mutants even before cell stimulation, and exceeded the WT levels after stimulation for at least 8 min. R552G displayed especially high translocation levels. Mutations that cause a defect in the REM domain (REM(−)) diminish but do not completely inhibit the cell-surface translocation of WT SOS^23^ (Fig. 1e). When the REM(−) mutation was introduced, the translocation of M269R and R552G was reduced. M269R with REM(−) (M269R/REM(−)) displayed a similar level of translocation as WT/REM(−). The translocation of R552G/REM(−) decreased but was still higher than that of WT/REM(−). In contrast, the translocation of R1131K was only slightly affected by REM(−) (Fig. 1e). These mutation-specific behaviours indicate that different mechanisms cause the hypertranslocation of the three NS mutants.

### Association rate constants of the NS mutants with cell membrane components

**Figure 2:**
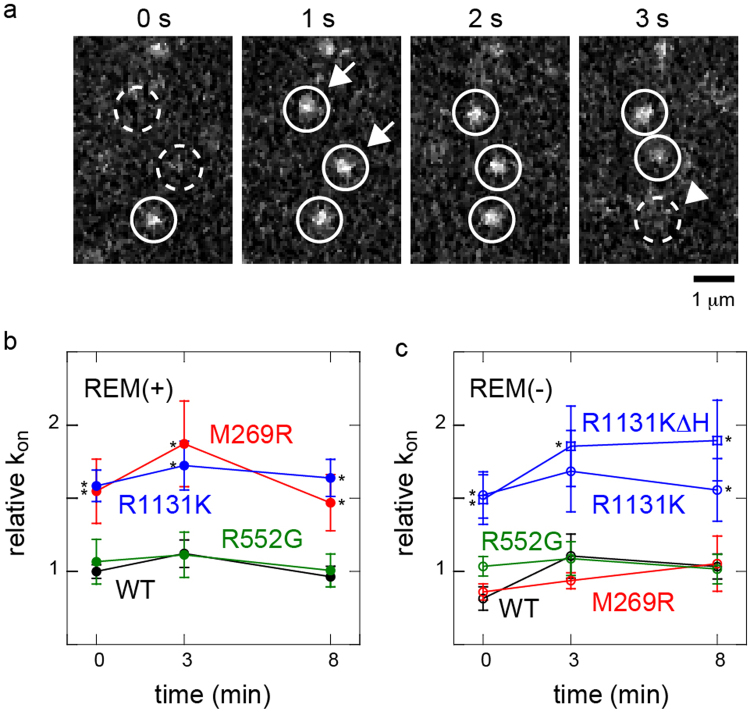
Association rate constants of NS SOS protein molecules with membrane components. (**a**) Successive images on the cell surface showing the appearance (arrows) and disappearance (arrowhead) of single SOS molecules. (**b**,**c**) Relative association rate constants (k_on_) of SOS molecules with the cell surface. The molecules contained the REM(−) mutation (**c**) or did not (**b**). R1131KΔH contained the REM domain but the H domain was deleted. The appearance rate of fluorescent SOS molecules per unit area of the cell surface was normalized to the fluorescence intensity in the cytoplasm and plotted as values relative to that of WT SOS before EGF stimulation (time 0). Averages of 5–10 cells with SE. Asterisks indicate statistical significance relative to the densities in WT or WT REM(−) at the indicated times (p < 0.05 on Mann–Whitney test).

In principle, the densities of cytoplasmic proteins on the cell surface are determined by their association and dissociation reactions with the plasma membrane components. If we assume a simple mass action reaction, the second-order association rate constant between a cytoplasmic protein and the cell surface is proportional to the appearance rate of single molecules in a unit area of the cell surface, after normalization to the concentration in the cytoplasm. Here, the concentration of a fluorescently labelled protein is proportional to the fluorescence intensity observed in the cytoplasm. We measured the membrane association rate constants of the SOS mutants based on these principles (Fig. 2a). The relative values of the association rate constants for M269R and R1131K were larger than that for WT, before and up to 8 min of EGF stimulation, whereas R552G showed a similar rate constant to that of WT (Fig. 2b). The large association rate constants of M269R and R1131K increased the membrane affinity of these NS mutants.

REM(−) decreased the association rate constant of M269R to a level similar to that of WT/REM(−) (Fig. 2c). Our previous study suggested that in WT SOS, the H and G domains determine the association rate constant with the plasma membrane, but the REM domain does not obviously affect the association^23^. However, in M269R the interaction between the REM domain and RAS affected the association rate constant, although M269 is located outside the REM domain. The association rate constant of R1131K was not affected by the REM(−) mutation (Fig. 2c). The deletion of the H domain without the REM(−) mutation (R1131KΔH) did not reduce the association rate constant (Fig. 2c)¸ which differs from our previous result for WT carrying the same deletion^23^. The increased association rate constant of R1131K with the plasma membrane that was caused by the mutated G domain probably concealed the role of the H domain in the initial association. As shown here, the mechanisms by which the association rate constant is increased differ for M269R and R1131K.

### Dissociation kinetics of NS mutants

**Figure 3:**
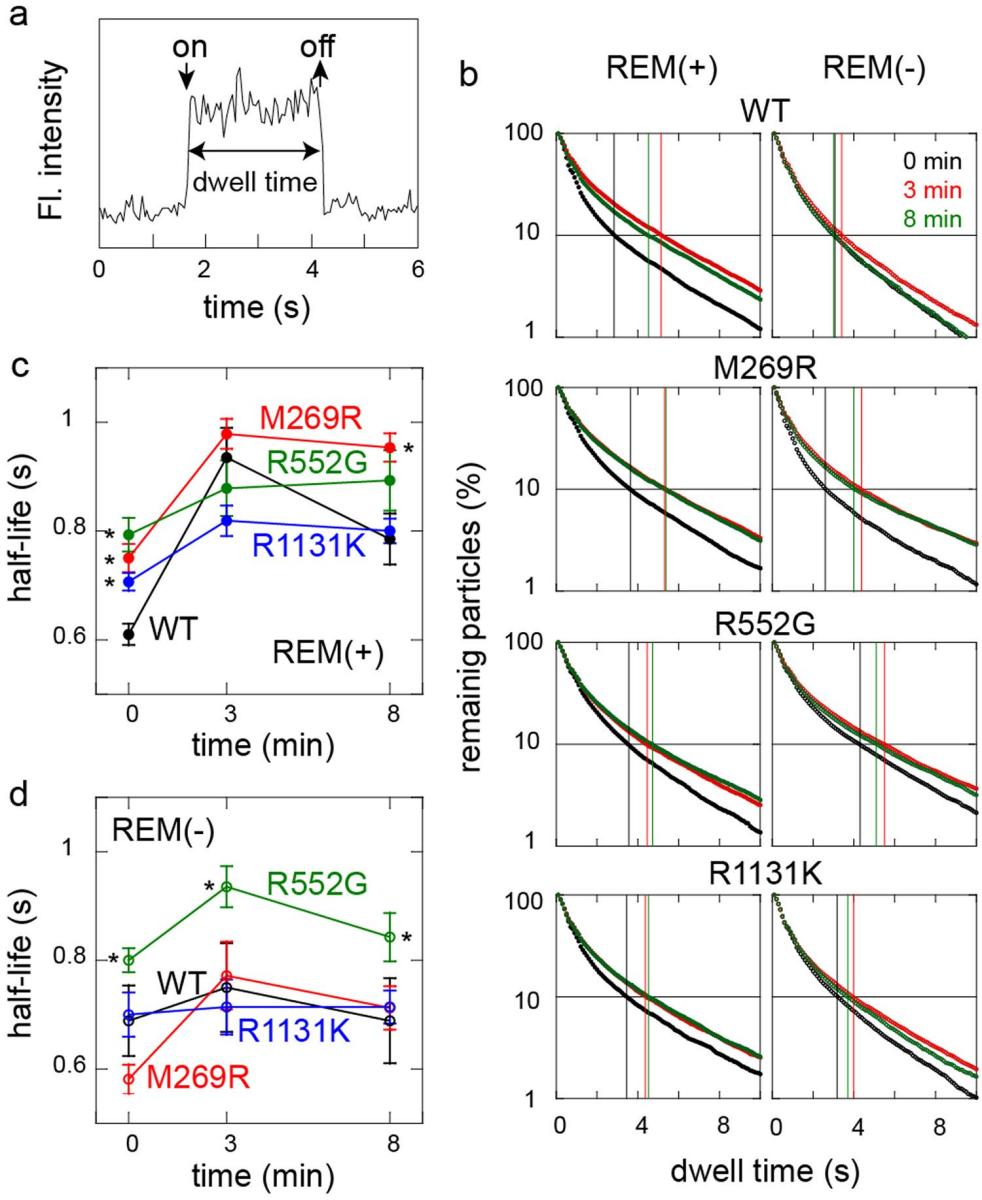
Dwell time distributions of NS SOS mutants on the cell surface. (**a**) Example of the change in fluorescence intensity on the cell surface, showing the association (on) and dissociation (off) of a single SOS molecule. (**b**) Release curves of SOS molecules from the cell surface. Molecules contained the REM(−) mutation (left) or did not (right). A total of 624–7222 molecules were observed under each condition. Black, red, and green plots show the curves obtained after stimulation with EGF for 0, 3, and 8 min, respectively. The vertical lines show the dwell times at the 10% remaining points. (**c**,**d**) Half-lives of the SOS molecules on the cell surface were plotted as indicators of the dissociation kinetics. SOS molecules contained the REM(−) mutation (**d**) or did not (**c**). Average of 7–10 cells shown with SE. Asterisks indicate a significant increase (p < 0.05 on Mann–Whitney test) compared with the WT or WT/REM(−) under the corresponding conditions.

The dwell times of single SOS protein molecules on the cell surface were measured to determine the dissociation kinetics from the plasma membrane components (Figs 2a and 3a). The release curves of the SOS molecules, reconstructed from the single-molecule dwell times, show complex shapes for all the NS mutants and WT (Fig. 3b), probably because the dissociation process involves the multiple membrane-association domains of SOS^23^. The half-life of the membrane association (median dwell time) was taken as a simple indicator of the dissociation kinetics (Fig. 3c,d). Because the average dwell time is strongly affected by a small population of very long dwell times, the half-life is a more robust parameter to reflect the quantitative properties of the complex dissociation kinetics as a whole. A longer half-life for the membrane association (i.e., a smaller effective dissociation rate constant) can explain a higher affinity for the cell surface.

Before cell stimulation, all the NS molecules displayed half-lives longer than that of WT (Fig. 3b,c). EGF stimulation caused the dwell time of WT SOS to increase to at least 8 min^23^. Similar increases were also observed for the NS SOS molecules. The half-lives after stimulation for 3 min were almost identical among all the SOS molecules, including WT. After stimulation for 8 min, M269R displayed a significantly longer half-life than those of the other molecules. The effects of the REM domain on the dissociation kinetics was examined by introducing an additional REM(−) mutation (Fig. 3d). As observed previously^23^, the half-life of WT/REM(−) was shorter than that of WT after EGF stimulation. The half-life of M269R/REM(−) was similar to that of WT/REM(−), indicating that the longer dwell time of M269R relative to that of WT was produced by the REM domain. The half-life of R552G/REM(−) was still longer than that of WT/REM(−) at every stage, indicating that this increase was independent of REM. R1131K/REM(−) had a half-life similar to that of WT/REM(−).

### RAF translocation in cells with NS SOS mutations

**Figure 4:**
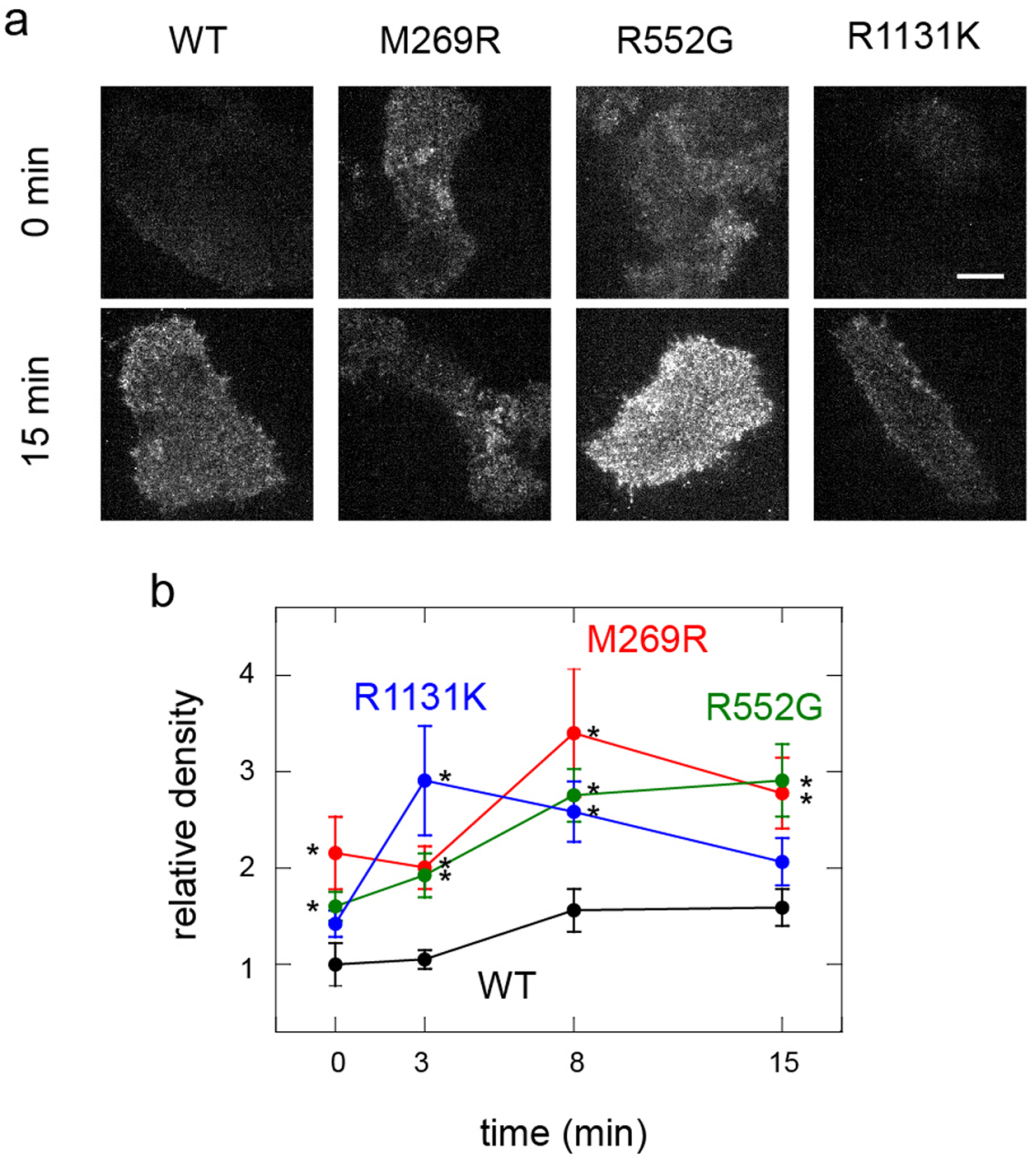
RAF translocation in cells expressing NS SOS mutants. (**a**) Typical micrographs show the cell-surface density of RAF molecules before (0 min) and after EGF stimulation for 15 min. Transfected cells expressed the indicated SOS molecules and GFP–RAF simultaneously. GFP–RAF on the basal cell surface was observed with a TIRF microscope. Bar: 20 μm. (**b**) Time courses of RAF translocation in cells expressing SOS and GFP–RAF molecules. Densities of GFP–RAF molecules on the cell surfaces were normalized to the intensity in the cytoplasm. The values were further normalized to that of cells expressing WT SOS before EGF stimulation (time 0). Average of 7–12 cells with SE. Asterisks show statistical significance relative to the densities in WT at the indicated times (p < 0.05 on Mann–Whitney test).

The translocation of RAF molecules from the cytoplasm to the plasma membrane that is caused by association with the RAS-GTP was monitored in living cells expressing the NS SOS mutants (Fig. 4a). A low level of RAF translocation to the cell surface was observed in resting cells because of the weak association between RAF and RAS–GDP^24–26^, and its association with the basal amount of RAS–GTP. In cells expressing WT SOS, an increase in the density of RAF on the cell surface was observed after EGF stimulation for 8 min, and this increase persisted for 15 min (Fig. 4b). This result is consistent with previous reports that RAS activation peaked at about 10 min^17^. Cells expressing M269R and R1131K SOS showed significantly higher density of RAF on their surfaces before EGF stimulation compared with that observed on cells expressing WT SOS. After EGF stimulation, all three NS SOS mutants, including R552G, induced the hypertranslocation of RAF compared with that in WT. Hypertranslocation indicates that under these conditions the NS SOS mutants caused the excessive activation of RAS, as expected.

## Discussion

The discovery of various genetic mutations in NS patients suggests that the common feature of NS (i.e., the excessive activation of the RAS–MAPK pathway) results from many molecular mechanisms. More than 30 different NS-related point mutations have been reported in the SOS molecule alone^21^. Interestingly, most of these do not occur in the Cdc25 domain, which contains the GEF activity of SOS. One highly probable mechanism by which these indirect mutations cause NS is by increasing the affinity of SOS for the plasma membrane before and/or after cell stimulation, because RAS is predominantly located on the plasma membrane and the GEF activity of SOS is regulated through the translocation of SOS from the cytoplasm to the cell surface. We found that the cell-surface densities were elevated for all three NS mutants investigated in this study, both before and after EGF stimulation, which induces SOS translocation (Fig. 1). A higher density of SOS on the cell surface simply increases the probability of it contacting the inactive form RAS, which is then activated by SOS.

However, it is possible that NS SOS activity is not simply determined by the density of SOS on the cell surface. Our results indicate that the mechanisms that induce excessive affinity for the cell surface are specific for each NS mutant. Membrane affinity is determined by the balance between association and dissociation. The observation of single SOS molecules revealed that M269R and R1131K have higher association rate constants with the plasma membrane components than WT, whereas R552G does not (Fig. 2). In contrast, before cell stimulation the dwell time of single SOS molecules on the cell surface, which reflects the dissociation rate constant, was longer for all three NS mutants than for WT (Fig. 3). After the cells were stimulated with EGF, the dwell time increased for all the SOS molecules, including WT, by up to 8 min. The loss of function of the REM domain, REM(−), diminished the effects of M269R in both the association and dissociation kinetics of SOS. However, the abnormalities in the dissociation kinetics of R552G and in the association kinetics of R1131K were independent of the REM(–) mutation. Therefore, these NS mutants differ in the characteristics of their membrane interaction kinetics and the dependence of these kinetics on the REM domain, although all three mutants commonly cause the hypertranslocation of SOS to the cell membrane.

**Figure 5:**
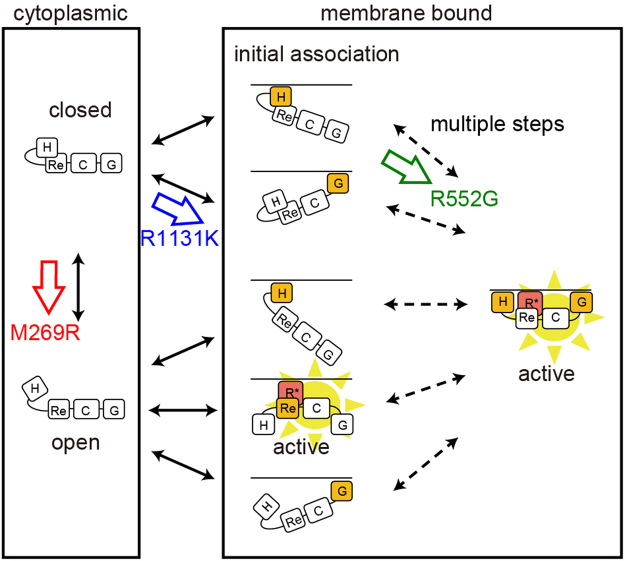
Schematic models of NS SOS interactions with the plasma membrane. An open/closed equilibrium is the basic structural dynamics of SOS, in which the closed form is the autoinhibitory state. In the cytoplasm, the equilibrium in WT SOS is highly biased toward the closed form. The initial association of WT SOS with the plasma membrane after EGF stimulation depends mainly on the H and G domains. Simultaneous associations of multiple domains with the membrane components then switch SOS into the open form, activating SOS through the positive feedback loop with RAS–GTP^23^. In the open form, the REM domain of SOS binds with RAS–GTP to establish the SOS–RAS positive feedback loop. Our results suggest that each NS mutations in SOS shifts the equilibrium toward SOS activation at specific positions in the scheme, as indicated. H, Re, C, G, and R* indicates the H domain, REM domain, Cdc25 domain, G domain, and RAS–GTP, respectively.

The changes in the interactions between each SOS mutant and the membrane components suggested by the single-molecule measurements are summarized in Fig. 5. In WT SOS, the initial association with the cell surface is explained with a model in which the H and G domains interact independently with the corresponding membrane components, acidic lipids, and the phosphorylated EGF receptor (via GRB2), respectively^23^. After the initial association, the H and G domains associate simultaneously with the membrane in a certain probability to form an intermediate state^23^. The REM(−) mutation destabilizes the intermediate state probably because it prohibits the interaction of SOS with RAS–GTP, but does not reduce the association rate constant of WT with the plasma membrane^23^. However, in M269R SOS the REM domain contributes not only to the stability of the association state (small dissociation rate constant with the membrane), but also to the initial association with the cell surface (large association rate constant with the membrane). In the crystal structure of the inactive form of WT SOS, the intramolecular interaction between the H domain and HL prevents the REM domain from interacting with the RAS molecule^15,20^. The REM domain in WT SOS seems to be exposed to RAS only after the molecule takes a specific membrane-associated conformation (i.e. the intermediate state) using the H and G domains simultaneously^23^.

The REM-dependent increase in the association rate constant suggests that the REM domain of M269R in the cytoplasm is exposed to RAS before SOS associates with the cell surface, and binds directly to RAS on the membrane. The enhanced interaction between the REM domain and RAS–GTP amplifies the activity of RAS through the SOS–RAS positive feedback loop.

R552 interacts with D140 and D169 in the H domain, maintaining the autoinhibitory structure of SOS^15^, which should be destabilized by the R552G mutation. However, the single-molecule kinetics suggest that unlike M269R, R552G adopts an active conformation only after its association with the membrane; i.e., R552G in the cytoplasm does not display an increased association rate constant for the plasma membrane. The reduced dissociation rate constant of R552G can be explained as a conformational preference that allows the simultaneous association of the H and G (and other) domains with the membrane components. In other words, R552G increases the probability of the open SOS conformation on the cell surface. In the open conformation, the probability of the interaction between the REM domain and RAS–GTP increases, thereby increasing the GEF activity. In WT and the other NS mutants investigated in this study, the REM/RAS–GTP interaction stabilized the membrane association state of SOS. However, in R552G the interaction between RAS and the REM domain did not determine the dissociation kinetics. A high probability of an open conformation will increase the REM/RAS–GTP interaction, inducing the hyperactivation of RAS by R552G, as suggested by the increased translocation of RAF in cells expressing R552G.

The G domain of SOS is the GRB2-binding site, and four serine residues in the G domain (S1132, S1167, S1178, and S1193) are phosphorylated by ERK in the negative feedback to SOS activity^27^. S1134 and S1161 in the G domain are also phosphorylated by RSK, which is regulated by ERK, inducing the downregulation of ERK activity^28^. These serine residues are in the proline-rich region of the G domain, which forms the complex with GRB2. Because R1131 is located near these serine residues, it is possible that changes in the formation of the SOS–GRB2 complex explain the dysregulation caused by R1131K. The positive charge on lysine may neutralize the effect of the negative charges introduced by serine phosphorylation. In our experiment, the increased association rate constant of R1131K was largely explained by the effect of the G domain (Fig. 2c), consistent with the possibility that a strong complex formed between R1131K and GRB2. Once the SOS molecule had associated with the cell surface, the dissociation kinetics of R1131K and their REM-domain dependence were similar to those of WT. The slight increase in the dwell time before cell stimulation can be attributed to the smaller late dissociation constant from the plasma membrane of the G domain relative to that of the H domain^23^. It is likely that the hyperactivation caused by R1131K is produced by a higher membrane association density, and not by the abnormal behaviour of single R1131K molecules after their association with the plasma membrane.

The positive feedback loop between SOS and RAS is critical for the activation of downstream molecules^17^. We observed that the REM(−) mutation largely prevented the translocation of RAF, a typical effector of RAS, from the cytoplasm to the cell surface for activation^23^. Because the feedback response is nonlinear, involving the product of the SOS–RAS reaction (RAS–GTP), small differences in the reaction conditions will be amplified, resulting in large differences in the output. The differential properties of the three NS mutants in inducing the hyperassociation of SOS with the cell surface may cause mutant-specific dynamics of RAS activation, which cannot be explained simply by the cell-surface density of the SOS molecules. For example, although the density of SOS on the cell surface was higher for R552G than for M269R (Fig. 1c), the basal translocation of RAF was higher with M269R than with R552G (Fig. 4b). This might be attributable to the more effective formation of the feedback loop in M269R, based on the enhanced interaction between the REM domain and RAS–GTP. At present, the relationships between the positions of the genetic mutations and the pathological NS phenotypes are unclear, but the prevalence of foetal macrosomia is reportedly higher in patients with mutations in the mutant class that includes M269R than in the class that includes R552G^21^. Therefore, it is important to clarify how these differences in molecular behaviour result in different pathologies.

As observed in NS, the pathology of many genetic diseases is multimodal and their symptoms are sometimes ambiguous. It is highly likely that the mutations involved in these diseases do not cause direct changes in the enzymatic activity of the mutant protein, but slightly dysregulate the protein dynamics and its molecular interactions. The single-molecule analysis of molecular behaviours in living cells can provide a useful assessment of the functions of such ambiguous mutations.

## Methods

### Preparation of plasmids

The construction of the cDNAs of Halo7-WT SOS and Halo7-REM(−) SOS has been described previously^23^. Halo7 was used to tag the N-terminus of SOS. The NS point mutations were introduced into Halo7-WT SOS with the QuikChange Lighting Site-Directed Mutagenesis Kit (Agilent Technologies) and PrimeSTAR® Max DNA Polymerase (Takara). The plasmid encoding GFP–RAF was constructed as reported previously^24^.

### Preparation of cells

All experiments were performed in HeLa cells (American Type Culture Collection). The cells were maintained in Dulbecco’s modified Eagle’s medium (DMEM) supplemented with 10% foetal bovine serum (FBS) at 37 °C under 5% CO_2_. The cells on coverslips were transfected with cDNAs using Lipofectamine® LTX with Plus™ Reagent (Invitrogen) 2 days before the experiments. After transfection, the cells were starved for 16 h in minimal essential medium (MEM) without serum or phenol red, and supplemented with 1% bovine serum albumin (BSA). To observe the SOS protein, the cells were stained with 100 nM HaloTag® TMR Ligand (Promega) for 15 min immediately before the experiments and washed.

### Single-molecule imaging and analysis

Single SOS molecules in the cells were observed with a home-made total internal reflection fluorescence microscope (TIRFM), based on an inverted microscope (IX83, Olympus) equipped with an objective PlanApo 60× NA 1.49 (Olympus). A 555 nm solid-state laser (GCL-075-555, CrystaLaser) was used for illumination, and fluorescent movies were acquired with a CMOS camera (ORCA-Flash4.0, Hamamatsu) at a frame rate of 20 s^−1^. After 3 × 3 pixel averaging and background subtraction with MetaMorph (Molecular Devices) and Image J (the National Institutes of Health), single molecules were detected and tracked with in-house software^29^ and TrackMate^30^. The dissociation kinetic analysis and statistical analysis were performed with Matlab (The MathWorks) and Origin (Originlab) as described previously^31^. The photo-bleaching rate constant of TMR conjugated to SOS (0.05 s^−1^; Nakamura *et al*.)^23^ was significantly smaller than the apparent values (several per second) of the dissociation rate constants of SOS from the plasma membrane components (Fig. 3). Thus, although the values of the half-life time of SOS included the effect of the photo-bleaching, contribution of the photo-bleaching to the estimated half-life time of SOS is not significant. To estimate the relative expression level of SOS in the cytoplasm, epi-fluorescence intensities of the Halo-tagged SOS were measured in the epi-illumination mode after observing the single SOS molecules on the plasma membrane in the TIR illumination mode in the same microscope as described previously^31^. All TIRFM studies were performed at 25 °C.

### Immunoblotting analysis

HeLa cells transfected with plasmids encoding the SOS molecules were incubated overnight in MEM, supplemented with 1% BSA. The cells were washed twice with Hank’s balanced salt solution (HBSS) and harvested in SDS solubilisation buffer. The proteins in the cell lysates were separated according to their molecular sizes on 10% or 8% polyacrylamide gel, and transferred to polyvinylidene difluoride membranes (BD Biosciences). The membranes were incubated in 5% skim milk with an anti-SOS1 antibody (#5890; Cell Signaling), anti-ERK antibody (#4696; Cell Signaling), or anti-pERK antibody (#9106; Cell Signaling). After the membranes were washed tree times with PBS, the membranes were incubated with a secondary antibody conjugated with alkaline phosphatase (Vectastain ABC-AP Kit; Vector Laboratories) or with horseradish peroxidase (#7074, #7076; Cell signalling). Antibody bindings were visualized with BCIP/NBT Color Development Substrate (Promega) or with ECL Prime Western Blotting Detection Reagent Kit (GE Healthcare).

## Electronic supplementary material

Supplementary Information
