# Supplementary material for: Mutation-Specific Mechanisms of Hyperactivation of Noonan Syndrome SOS Molecules Detected with Single-molecule Imaging in Living Cells
Source: Sci Rep. 2017 Oct 26;7:14153. doi: 10.1038/s41598-017-14190-6 (PMC5658395; doi:10.1038/s41598-017-14190-6)
Supplement: Supplementary file 1 — Supplementary Information [file 41598_2017_14190_MOESM1_ESM.pdf]

Supplementary Information

Mutation-Specific Mechanisms of Hyperactivation of Noonan Syndrome SOS Molecules Detected with Single-molecule Imaging in Living Cells

Yuki Nakamura[1], Nobuhisa Umeki[1], Mitsuhiro Abe, and Yasushi Sako*

Cellular Informatics Laboratory, RIKEN, 2-1 Hirosawa, Wako 351-0198, Japan

[1]These authors contribute equally to this work
*corresponding author: sako@riken.jp

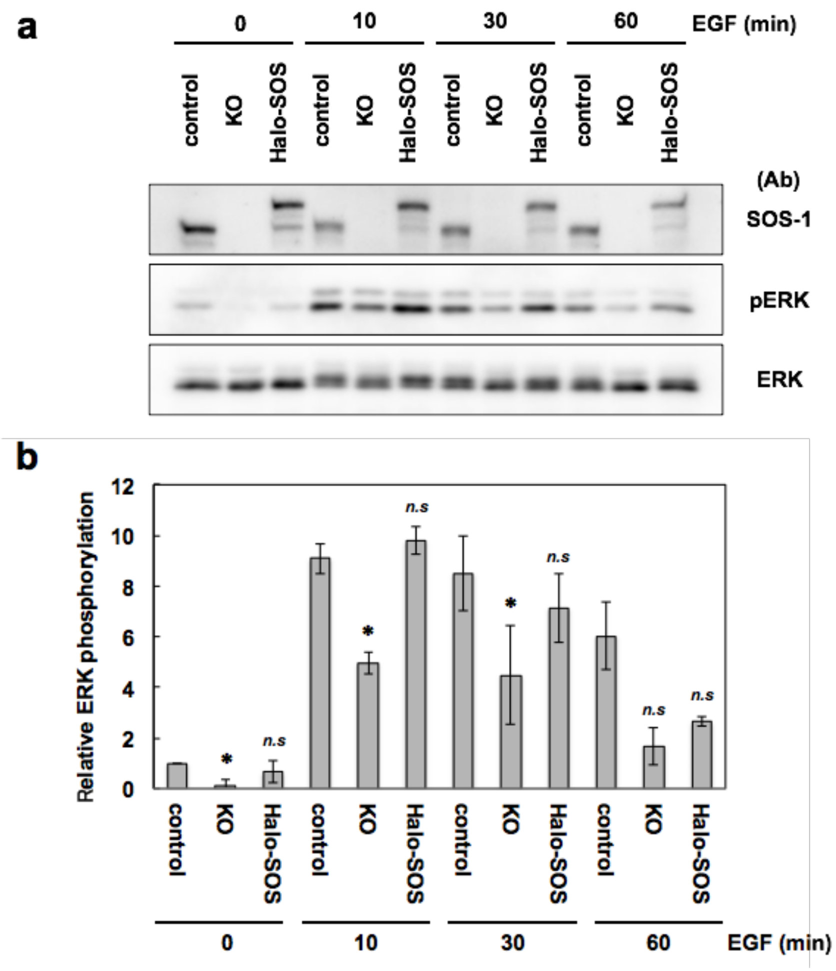

Supplementary Figure S1. Halo-tag does not influence SOS function

a. ERK phosphorylation after the EGF stimulation was examined in parental (control), SOS1 knockout (KO), and SOS1 KO/Halo7-WT SOS1 knock-in (Halo-SOS) HeLa cells. Cell extracts were analyzed by SDS-PAGE and immunoblotting with antibodies (Ab) against indicated antigens. Endogenous SOS2 in all cells were knocked-down by RNA interference before experiment (see Supplementary Methods). EGF-induced ERK phosphorylation in KO cells was significantly lower than that in the control cells, whereas this reduction was rescued in Halo-SOS cells.

b. Quantification of relative ERK phosphorylation level. ERK phosphorylation levels were normalized to the amounts of total ERK expression. Average of ERK phosphorylation levels in four independent experiments are shown as the relative value to that in control cells before stimulation (time 0). Error bars show standard error. Asterisks denote statistical significance compared with the control cells at the indicated times (*$p<0.05$ on *t*-test). *n.s*; not significant. The result indicated that Halo-tag moiety of the fusion protein did not influence the SOS functions.

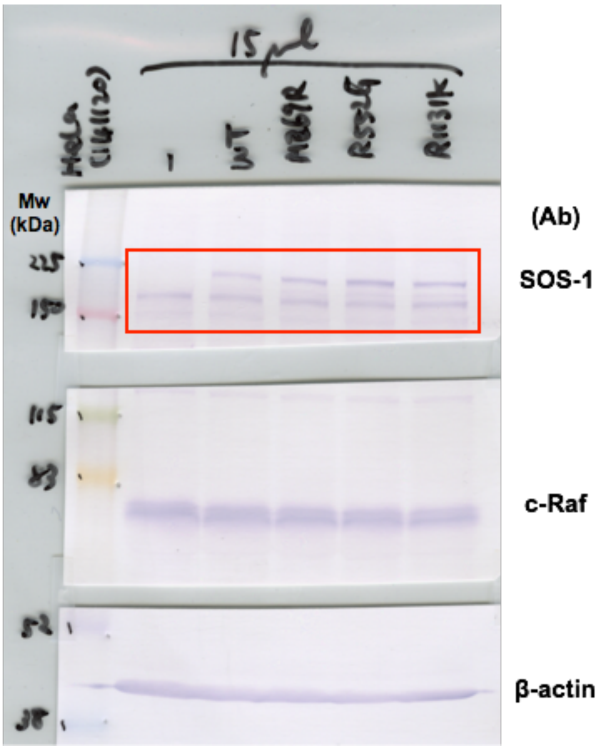

Supplementary Figure S2. The full-length image of immunoblot shown in Figure 1b

Expression of Halo-tagged SOS, endogenous SOS, c-RAF, and β-ACTIN, assessed with a immnoblotting analysis of cell lysates with the indicated antibodies (Ab). The left-most lane shows the molecular weight markers (Mw). Boxed area was cropped for the image of Fig. 1b.

Supplementary Methods

Generation of SOS1 knockout cells using CRISPR/Cas9 gene editing

To construct the gene editing plasmid, DNA oligomers (3'-caccgTTTTTCCAGTCAATAGGCGA-5' and 3'-aaacTCGCCTATTGACTGGAAAAAc-5') were synthesized, annealed, and cloned into the PX459 vector (#48139, Addgene) at the *Bbs*I site as previously described [1]. The resultant plasmid was transfected into HeLa cells using Lipofectamine 3000 (Thermo Fisher Scientific). Single colonies were selected after a 0.2 μg/ml puromycin selection for 3 days. Genomic DNAs were extracted from the clones using GenElute™ Mammalian Genomic DNA Miniprep Kit (Sigma-Aldrich). Genome sequences were examined by PCR using the following primers: 5'-AATTGTGCTCGCATAGTCGTGCCCC-3' and 5'-CCCTTATCCTCACACTGAGTCCCTGAGTC-3'.

Simultaneous integration of Halo7-WT SOS1 and knockout of SOS1

Integration of Halo7-WT SOS1 to the SOS1 locus in HeLa cells was based on homology-independent targeted integration (HITI) strategy [2]. To construct the plasmid for integration, silent mutations were introduced at the Cas9/gRNA target sequence (TTTTTCCAGTCAATAGGCGA) in the Halo7-WT SOS plasmid, and another Cas9/gRNA target sequence was reversely introduced before the promoter of Halo7-WT SOS in the plasmid. To integrate Halo7-WT SOS1 to the SOS1 locus and to knock out SOS1 simultaneously, both plasmids for integration and for gene editing were transfected into HeLa cells. Single colonies were selected after puromycin treatment as described above. After genome sequences were examined, expressions of Halo7-WT SOS1 were checked by immunoblotting analysis, and cell lines expressing Halo7-WT SOS1 were selected (Supplementary Fig. S1a).

Knockdown of SOS2 and ERK phosphorylation assay

The siRNA for SOS2 was designed according to the online program at http://rnai.co.jp/lsci/about.html. The sequences were as follows, sense: 5′-CUGUUUACGGAUAGAACCAGA-3′, anti-sense: 5′-UGGUUCUAUCCGUAAACAGUA-3′. Synthesized siRNA was transfected using Lipofectamine RNAiMAX (Thermo Fisher Scientific) into SOS1 knockout cells, Halo7-WT SOS1 expressing cells, and intact HeLa cells. After the transfection, cells were maintained 2 days in DMEM supplemented with 10% FBS at 37 °C under 5% CO_2, then starved for 16 h in MEM without serum and supplemented with 1% BSA. To induce ERK phosphorylation, the

cells were treated with or without 10 ng/ml EGF (Sigma-Aldrich) at 37 °C and the reactions were stopped by addition of SDS-sample buffer, and then the samples were subjected to immunoblotting analysis.

Supplementary References

[1] Ran, F.A., Hsu, P.D., Wright, J., Agarwala, V., Scott, D.A. & Zhang, F. Genome engineering using the CRISPR-Cas9 system. *Nat Protoc* **8**, 2281-2308 (2013).

[2] Suzuki, K., Tsunekawa, Y., Hernandez-Benitez, R., Wu, J., Zhu, J., Kim, E.J., Hatanaka, F., Yamamoto, M., Araoka, T., Li, Z. et al. In vivo genome editing via CRISPR/Cas9 mediated homology-independent targeted integration. *Nature* **540**, 144-149 (2016).
